# High Circulating Sonic Hedgehog Protein Is Associated With Poor Outcome in EGFR-Mutated Advanced NSCLC Treated With Tyrosine Kinase Inhibitors

**DOI:** 10.3389/fonc.2021.747692

**Published:** 2021-12-14

**Authors:** Paul Takam Kamga, Aurélie Swalduz, Adrien Costantini, Catherine Julié, Jean-François Emile, Maurice Pérol, Virginie Avrillon, Sandra Ortiz-Cuaran, Pierre de Saintigny, Etienne Giroux-Leprieur

**Affiliations:** ^1^ Université Paris-Saclay, UVSQ, EA 4340 BECCOH, Boulogne-Billancourt, France; ^2^ Department of Medical Oncology, Centre Léon Bérard, Lyon, France; ^3^ Univ Lyon, Université Claude Bernard Lyon 1, INSERM 1052, CNRS 5286, Centre Léon Bérard, Centre de Recherche en Cancérologie de Lyon, Lyon, France; ^4^ Department of Respiratory Diseases and Thoracic Oncology, APHP—Hopital Ambroise Pare, Boulogne-Billancourt, France; ^5^ Department of Pathology, APHP—Hopital Ambroise Pare, Boulogne-Billancourt, France

**Keywords:** non-small cell lung cancer (NSCLC), epidermal growth factor receptor (EGFR), Sonic Hedgehog (Shh), biomarker, tyrosine kinase inhibitor (TKI)

## Abstract

**Introduction:**

Growing preclinical evidence has suggested that the Sonic hedgehog (Shh) pathway is involved in resistance to tyrosine kinase inhibitor (TKI) therapy for EGFR-mutated (EGFRm) non-small cell lung cancer (NSCLC). However, little is known concerning the prognostic value of this pathway in this context.

**Materials and Methods:**

We investigated the relationship between plasma levels of Shh and EGFRm NSCLC patients’ outcome with EGFR TKIs. We included 74 consecutive patients from two institutions with EGFRm advanced NSCLC treated by EGFR TKI as first-line therapy. Plasma samples were collected longitudinally for each patient and were analyzed for the expression of Shh using an ELISA assay. The activation of the Shh–Gli1 pathway was assessed through immunohistochemistry (IHC) of Gli1 and RT-qPCR analysis of the transcripts of Gli1 target genes in 14 available tumor biopsies collected at diagnosis (baseline).

**Results:**

Among the 74 patients, only 61 had baseline (diagnosis) plasma samples, while only 49 patients had plasma samples at the first evaluation. Shh protein was detectable in all samples at diagnosis (*n* = 61, mean = 1,041.2 ± 252.5 pg/ml). Among the 14 available tumor biopsies, nuclear expression of Gli1 was observed in 57.1% (8/14) of patients’ biopsies. Shh was significantly (*p* < 0.05) enriched in youth (age < 68), male, nonsmokers, patients with a PS > 1, and patients presenting more than 2 metastatic sites and L858R mutation. Higher levels of Shh correlated with poor objective response to TKI, shorter progression-free survival (PFS), and T790M-independent mechanism of resistance. In addition, the rise of plasma Shh levels along the treatment was associated with the emergence of drug resistance in patients presenting an initial good therapy response.

**Conclusion:**

These data support that higher levels of plasma Shh at diagnosis and increased levels of Shh along the course of the disease are related to the emergence of TKI resistance and poor outcome for EGFR-TKI therapy, suggesting that Shh levels could stand both as a prognostic and as a resistance biomarker for the management of *EGFR*-mutated NSCLC patients treated with EGFR-TKI.

## Introduction

Lung cancer is the main cause of cancer death in the Western world. Non-small cell lung cancer (NSCLC) represents 85%–90% of lung cancer subtypes (1). Growing efforts have led to tremendous advances in early diagnosis and the implementation of more efficient drugs that target key oncogenic events (2). Among others, the introduction of epidermal growth factor receptor (EGFR)-tyrosine kinase inhibitors (EGFR-TKIs) as first-line therapy for the treatment of *EGFR*-mutated NSCLC patients has led to longer progression-free survival (PFS) and higher response rates compared to platinum-based chemotherapy (3–6). Notwithstanding the clinical advantage observed in patients treated with EGFR-TKI, primary or acquired resistance limits the benefit of TKI treatment (7, 8). Therefore, there is still a continual need of new predictive biomarkers for EGFR-TKI therapy. Studies have addressed many sources of EGFR-TKI resistance, underlining the involvement of EGFR-dependent (i.e., T790M or C797S resistance mutations) or EGFR-independent molecular aberrations (i.e., *MET* amplification or the activation of other oncogenic pathways) (9). In general, primary or acquired molecular aberrations involved in poor clinical outcome collaborate with pro-survival signaling such as Hedgehog, Wnt, Notch, Akt, and Ras/Erk (10–12). Therefore, the expression/activation levels of these pathways have been proposed as predictive biomarker for EGFR-TKI therapy.

Hedgehog (Hh) pathway is a highly conserved pathway involved in developmental processes such as tissue patterning and organogenesis (13). There are three hedgehog ligands, Desert Hedgehog, Indian Hedgehog, and Sonic Hedgehog (Shh). In human, the activation of the pathway occurs when the ligand binds to the receptor Patched (Ptch), activating the Ptch-coreceptor Smoothened (Smo). Smo stabilizes and activates the transcription factors of the Gli proteins family; then, Gli proteins enter into the nucleus to activate genes involved in cell proliferation, self-renewal, and survival during development or in cancer (14, 15). The overexpression or/and the hyperactivity of Shh/Gli signaling are found in several solid and hematological malignancies, being associated with aggressive disease, poor survival, and resistance to many anti-cancer therapies (16–19). In NSCLC, we have observed that the activation of Hedgehog pathway was associated with resistance to platinum-based chemotherapy and to immune checkpoint inhibitors (20, 21). In *EGFR*-mutated NSCLC, inhibition of the Shh pathway sensitizes primary tissues and cancer cell lines both to classic chemotherapy and EGFR-TKIs, suggesting a role for Hh pathway in resistance to EGFR TKI (22). Therefore, we hypothesized that the expression levels of Shh could predict the outcome of *EGFR*-mutated NSCLC patients treated with EGFR-TKIs. In this study, expression levels of Shh in plasma samples collected from patients treated with EGFR-TKIs were correlated with patients’ features to demonstrate that high levels of Shh in patient samples is a biomarker of poor prognosis.

## Materials and Methods

### Patients and Samples

Plasma samples and lung biopsies were collected as previously described (21, 23), from consecutive patients with advanced (not irradiable stage IIIb or stage IV) *EGFR*-mutated NSCLC treated by EGFR-TKIs between 08/2011 and 07/2019 at the Department of Respiratory Medicine and Thoracic Oncology (APHP—Ambroise Pare Hospital) and within the framework of the Centre Léon Bérard Cancer Center LIBIL (NCT02511288) (24, 25). All patients were included in the study after written informed consent, as approved by the two Institutional Review Boards (IRB) including the CPP IDF n°8 (ID CRB 2014-A00187-40) and the CPP Ouest 6 (ID-RCB: 2015-A00640-49). Lung biopsies (*n* = 14) were collected at diagnosis (diagnosis/baseline). Prospective consecutive collection of the plasma was made before the beginning of the TKI treatment, 6–8 weeks after the beginning of the TKI (first evaluation, *n* = 61), and at disease progression. All other collection points were referred to as follow-up.

### Enzyme-Linked Immunosorbent Assay

Plasma levels of Shh were assessed through ELISA assay as previously described (21, 26). Briefly, 50 µl of each plasma sample was loaded in duplicate into the 96-well Shh ELISA plate (ab100639, Abcam Cambridge, UK). After 2.5 h of incubation at room temperature (RT), wells were washed and probed for 1 h at RT with Biotinylated Shh detection antibody. Then, wells were washed and probed with HRP-Streptavidin for 45 min at RT. Wells were washed and the revelation was performed by adding in each well 100 µl of a TMB substrate following by the addition of a Stop solution. Optical densities at 450 nm were determined with a plate reader, the Multiscan GO reader, V.1.01.10 (ThermoFisher Scientific, France). The concentration of Shh in each sample was determined using standard controls (recombinant proteins) and the generated standard curve.

### Immunohistochemistry

Formalin-fixed paraffin-embedded tissue blocks were sectioned (4 μm) using a microtome, transferred to slides, and allowed to dry overnight. Samples were stained using a LEICA BOND-III automate. Activation of the Hh pathway was assessed through the staining of nuclear Gli1 (anti-Gli1 mouse monoclonal antibody sc-515751; 1:5; Santa Cruz Biotechnology, Santa Cruz, USA) in tumor cells. Stained slides were mounted on an optical microscope Axio ZEISS Scope. They were evaluated and scored by one biologist (PK) and one pathologist (J-FE). Gli1 nuclear staining was evaluated on tumor cells as recently described. A sample of Basal Cell human carcinoma (BCC) was used as a positive control and nonspecific immunoglobulin isotype was used as a negative control (21, 23).

### Reverse Transcription and Quantitative Polymerase Chain Reaction

RNA extraction from tumor tissue and reverse transcription using previously described TaqMan PCR primers and probes were performed as previously described (21). Briefly, RNA was extracted from tumor tissue using the AS1480 Maxwell RSC Simply RNA tissue kits (Promega, USA) according to the manufacturer’s instructions. One microgram of RNA was reverse transcribed in cDNA using the High-capacity RNA cDNA kit (ThermoFisher Scientific, France) and amplified using the TaqMan Universal PCR Master Mix, No AmpErase UNG (ThermoFisher Scientific, France). Primers and probes specific for Gli1 target genes and control gene β-actin (**Table S1**) were purchased commercially and used according to the manufacturer’s instructions (ThermoFisher Scientific, catalog number 4331182). All values were normalized to the control gene β-actin using the ΔΔ Ct method. Here, again, a BCC sample was used as positive control.

### Statistical Analysis

Statistical analysis was performed using GraphPad Prism 5 (La Jolla, CA, USA) and XLSTAT 2019.1.3 (Addinsoft, Paris, France). The Wilcoxon-Mann–Whitney and Kruskal–Wallis methods were used to compare two groups or more than two groups, respectively. Pearson’s Chi-squared analyses including the parametric Chi-squared and the non-parametric Fisher’s exact test were used to test the association between variables. Survival curves including overall survival (OS) and progression-free survival (PFS) were calculated by the Kaplan–Meier method and the application of log-rank test. *p*-values less than 0.05 were considered as significant.

## Results

### Patients

Clinical characteristics of patients are described in **Table 1**. Seventy-four consecutive patients were included in the study. The median age was 67 years old (range, 35 to 90 years old). Patients were mostly females (79.7%, *n* = 59) and nonsmokers (66.2%, *n* = 49). The main histological type was adenocarcinoma (95.9%, *n* = 71). Most patients had a stage IV disease (78.4%, *n* = 58) including 29 patients with central nervous system (CNS) metastasis (39.2%). Forty-seven (62.2%) NSCLC were Exon 19 mutated, 22 (29.7%) were L858R mutated patients, and 5 cases were found with another EGFR mutation type (**Table 1**). Only 61 patients with available plasma samples at diagnosis were included in correlative studies because 13 were excluded for missing data and/or no available sample at baseline (**Figure S1**). Among them, 20.1% (*n* = 23) were treated with erlotinib, 24.6% (*n* = 15) received gefitinib, 19.7% (*n* = 12) received afatinib, and 14.8% (*n* = 9) received osimertinib. Thirty-six patients (60.7%) responded to the therapy including 2 complete responses and 34 partial responses. On the other hand, 14 (22.9%) patients did not respond to the treatment, including 5 patients with stable disease and 6 patients in progression.

**Table 1:** Demographic and clinical profile of the study cohort.

Variable	Patients (*n* = 74)
Age	
Median [range]	67 [35–90]
<67	37 (50%)
>67	37 (50%)
Gender	
Female	59 (79.7%)
Male	15 (20.3%)
Smokers	
Never	49 (66.2%)
Former	17 (23.0%)
Current	6 (8.1%)
Unknown	2 (2.7%)
Performance Status	
0	17 (23.0%)
1	44 (59.4%)
2	8 (10.8%)
3	1 (1.4%)
Unknown	4 (5.4%)
Histology	
Adenocarcinoma	71 (96.0%)
Squamous cell carcinoma	2 (2.7%)
Large cell neuroendocrine carcinoma	1 (1.3%)
Number of metastatic sites	
0	4 (5.4%)
1	21 (28.4%)
2	13 (17.6%)
3	9 (12.2%)
4	9 (12.2%)
5	6 (8.1%)
Unknown	12 (16.2%)
CNS metastasis	29 (39.2%)

### Shh and Gli1 Expression at Baseline

**Figure 1:**
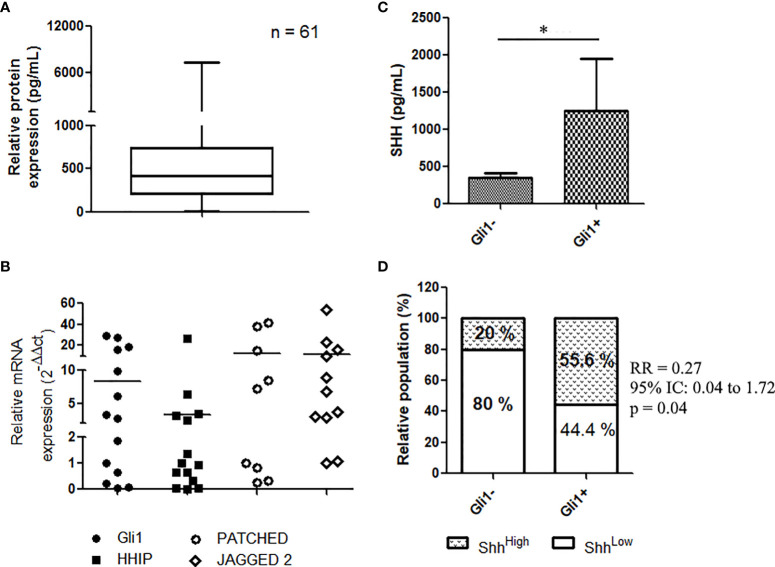
Shh signaling expression and activation in EGFR-mutated NSCLC patients: **(A)** Shh concentration in plasma from patients at diagnostic (*n* = 61) as assessed by ELISA assays. **(B)** mRNA expression of Gli1 target genes in NSCLC; data are expressed as mean ± SEM of 14 patients analyzed in duplicate. **(C)** Representative, IHC of Gli1 in NSCLC (*n* = 14). **(C, D)** Expression of Shh in plasma from patients (14) according to Gli1 expression. *p < 0.05.

To determine whether plasma levels of Shh were associated to patient outcomes, we first used the ELISA assay to analyze levels of circulating Shh in the plasma samples from NSCLC patients. Assessed in 61 samples collected before TKI initiation, the protein expression was readily found in plasma samples with a median of 466.185 pg/ml (range: 0.3–11,522.6 pg/ml) (**Figure 1A** and **Table S2**). The Kolmogorov–Smirnov (KS) normality test confirmed that data were normally distributed (*p* < 0.001) (**Table S2**).

We recently provided evidence that the Shh–Gli1 signaling axis is operative in NSCLC, demonstrating that primary lung biopsies from NSCLC express *GLI1* transcript and Gli1 target genes including *HHIP, PTCH*, and *JAG2* (20). Similarly, analyzing the mRNA in 14 patients’ tissues, we found that NSCLC expressed HHIP, PTCH, and JAG2 transcripts (**Figure 1B**), confirming that the Shh signaling is operative in NSCLC (**Figure 1B**). We then asked whether expression of plasma Shh was related to the pathway activation in NSCLC. Shh pathway activation was assessed by the mean of the nuclear staining through IHC of Gli1, in tumor tissue samples from 14 patients with known Shh plasma concentrations. Nuclear expression of Gli1 (**Figure S2**) was observed in 57.1% (8/14) of patients’ biopsies. Patients classified as Gli1-positive patients (Gli1+) showed elevated levels of plasmatic Shh compared to Gli1- patients (1,255 ± 696.1 vs. 352.9 ± 59.8; *p* = 0.05) (**Figure 1C**), suggesting an association between tumoral activation of the Shh pathway and the plasmatic concentration of its ligand. We applied a cutoff to divide plasma samples into low expressing samples (Shh < median, *n* = 34) and high expressing samples (Shh > median, *n* = 27). We used a Fisher’s rank test to demonstrate a positive association between the nuclear expression of Gli1 and higher levels of Shh in patients’ plasma [relative risk, RR (95% CI, confidence interval), 0.27 (0.041–1.73); *p* = 0.05] (**Figure 1D**).

### Correlation Between Shh Levels and Patients’ Characteristics

**Figure 2:**
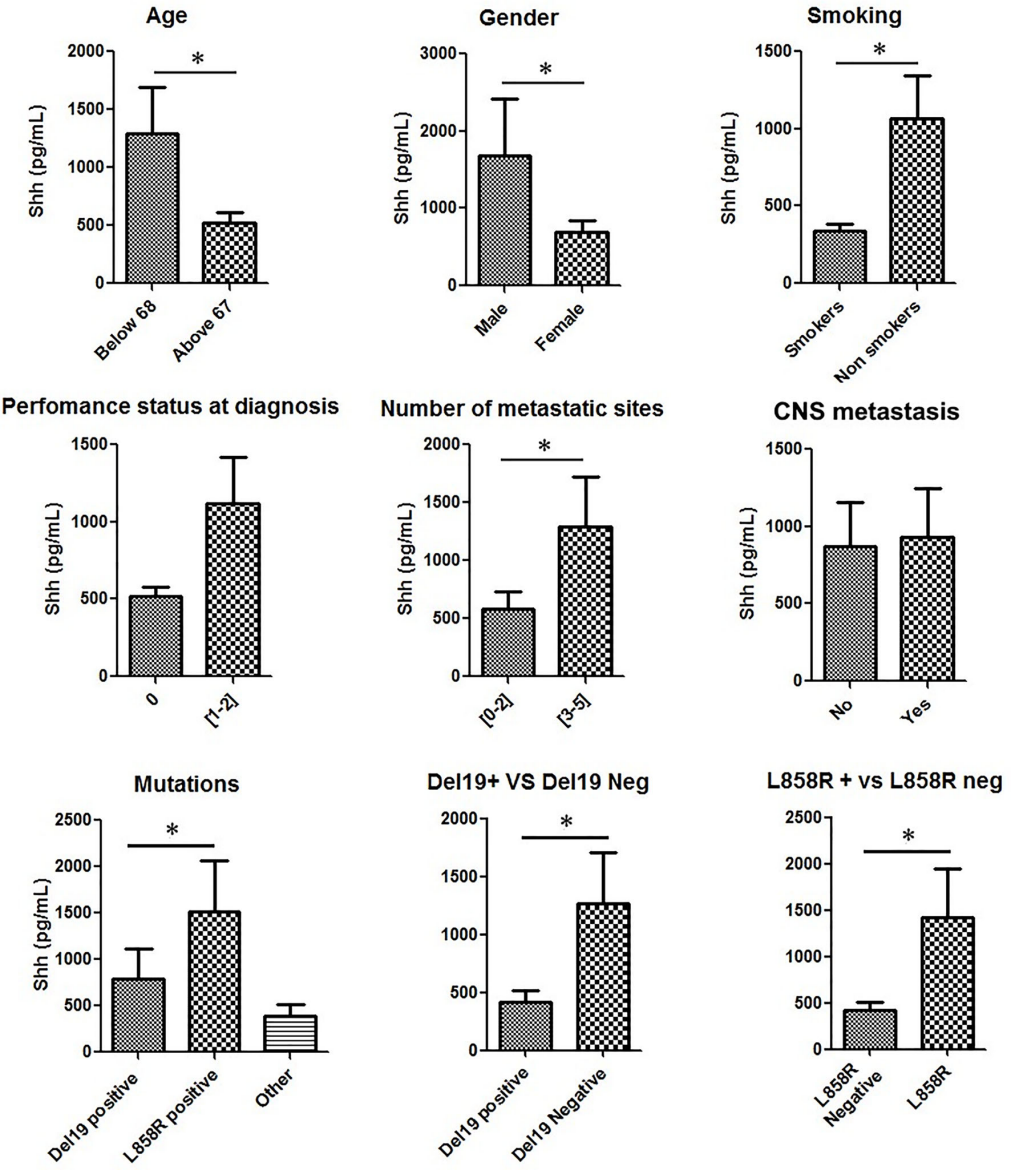
Shh concentrations in plasma from patients at diagnosis according to patients’ characteristics: Patient samples analyzed for Shh expression were classified according to age, gender, smoking status, performance status at diagnosis, number of metastatic sites, central nervous system (CNS) metastasis, and EGFR mutation type. A Mann–Whitney test was used to analyze the differences between means. *p < 0.05.

We then looked for associations between Shh plasma levels and patients’ characteristics. In accordance with previous studies, parameters considered were age, gender, smoking status, performance status (PS) at diagnosis, number of metastatic sites, CNS metastasis, and *EGFR* mutation type (**Figure 2** and **Table S3**) (23, 26, 27). Shh was significantly (*p* < 0.05) enriched in youth (age < 68 years old), males, and nonsmoker patients (**Figure 2**). However, we found no association between Shh levels and any of these three parameters (**Table S3**). A trend for higher Shh plasma levels in patients with a PS > 1 and those with more than 2 metastatic sites was observed (**Figure 2**), without reaching statistical significance (**Table S3**).

Then, we analyzed Shh levels in patients according to their specific *EGFR* mutation (del ex19, L858R and other mutations). Shh expression levels in these three groups were respectively 542.2 ± 94.29 pg/ml, 1,274 ± 440.4 pg/ml, and 524.8 ± 98.30 pg/ml (**Figure 2**). Compared to all other patients, patients with L858R *EGFR*-mutated NSCLC displayed higher Shh levels (**Figure 2**). A Pearson chi-squared analysis revealed a positive association between higher levels of Shh and L858R mutation [RR (95% CI), 0.4 (0.16–0.99); *p* = 0.02] and between del ex19 mutation and lower levels of plasmatic Shh [RR (95% CI), 1.55 (1.11–2.16); *p* = 0.02].

### Correlation With Treatment Response

**Figure 3:**
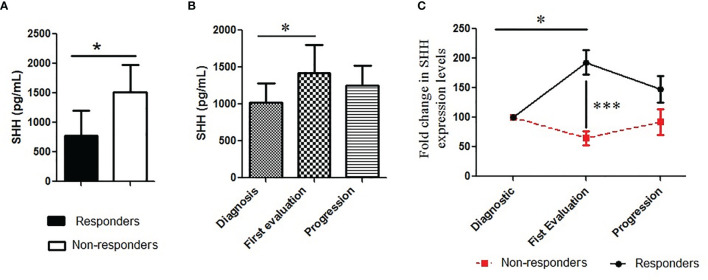
Pattern of plasmatic Shh concentration along the course of the disease: **(A)** Shh was analyzed in plasma collected from patients and classified according to **(A)** treatment response (responders vs. non-responders); **(B, C)** and treatment steps (diagnosis or base line, the first evaluation and the progression). A Mann–Whitney test was used to analyze the differences between means. *p < 0.05, ***p < 0.001.

Inhibition of Shh–Gli1/2 signaling is known to sensitize NSCLC to TKI treatment (10). Here, we have observed that Shh levels are related to patients’ characteristics such as the type of *EGFR* mutation, which is a well-known factor able to influence patients’ outcomes (28). It suggested that Shh plasma levels could vary along the disease course, being correlated to clinical response to TKI therapy. To validate this hypothesis, we first tested the relation between baseline Shh and response to TKI treatment. Shh analysis showed that the protein levels at baseline were lower in the plasma of responders compared to non-responders (779.4 pg/ml vs. 1,510 pg/ml; *p* = 0.01) (**Figure 3A**). Correlation test confirmed a positive association between lower levels of plasmatic Shh at diagnosis and a good response to therapy [RR (95% CI), 0.45 (0.22–0.90); *p* = 0.006]. The changes of Shh levels along the course of the disease were also evaluated. Considering all patients including responders and non-responders, we observed increasing levels of Shh in the plasma of patients at the time of first evaluation, with a median fold increase by 30% (**Figure 3C**). The separation of patients into responders and non-responders revealed that the variation in Shh levels was mainly observed in responders, while the non-responders showed a stable level of Shh, notwithstanding the disease steps (**Figure 3B**).

### Correlation With Patients’ Survival

**Figure 4:**
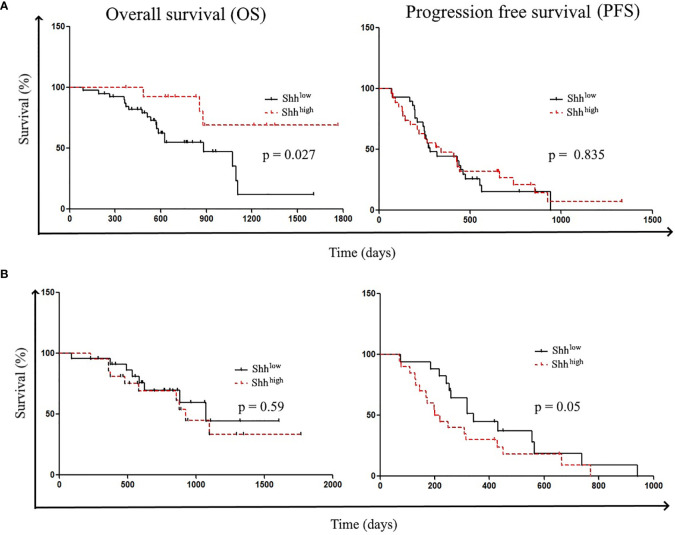
Patient’s survival according to Shh levels: Patient survivals were classified according to **(A)** Shh levels at diagnosis (Shh^low^ vs. Shh^High^) and **(B)** Shh levels at the first evaluation (Shh^low^ vs. Shh^High^).

Then, we analyzed patients’ survival. Shorter OSs were significantly related to the following parameters: the absence of del19 mutations [hazard ratio (HR) (95% CI), 0.28 (0.1–0.79); *p* = 0.0156] and age > 67 years [HR (95% CI), 0.29 (0.11–0.73); *p* = 0.01]. However, while older patients showed shorter PFS [HR (95% CI), 0.515 (0.28–0.95); *p* = 0.035], there was no difference in PFS according to del 19 mutations (**Table S4**). Shorter OS was also associated with the presence of L858R mutations [HR (95% CI), 2.5 (0.87–7.19)] and PS > 0 [HR (95% CI), 0.62 (0.18–2.07); *p* = 0.43] (**Table S4**). To study the influence of Shh levels on patients’ survival, patients were classified according to the Shh concentration; Shh^low^ (32/61) and Shh^high^ (29/61) 5/61 patients were excluded for clinical reasons. In accordance with the work of Kim et al., we found that patients with higher Shh levels at diagnosis showed prolonged OS compared to patients with lower concentrations [OS: undefined vs. 884 days; HR (95% CI), 3.12 (1.288–7.561); *p* = 0.0271] (**Figure 4A**) (29). However, preclinical data revealed that inhibition of Shh–Gli1/2 signaling is known to sensitize NSCLC to TKI treatment, supporting the fact that level of Shh–Gli1/2 signaling during TKI therapy could reflect or influence treatment outcome and patient survival (10). Thus, we also analyzed patient survival according to Shh levels in plasma samples at the first evaluation following TKI induction. Interestingly, patients with lower Shh levels at the first evaluation underwent longer PFS (342 vs. 210 days) compared to patients with high Shh levels (HR (95% CI), 0.578 (0.2831–1.180); *p* = 0.05) (**Figure 4B**).

### Correlation With Resistance Mechanisms

**Figure 5:**
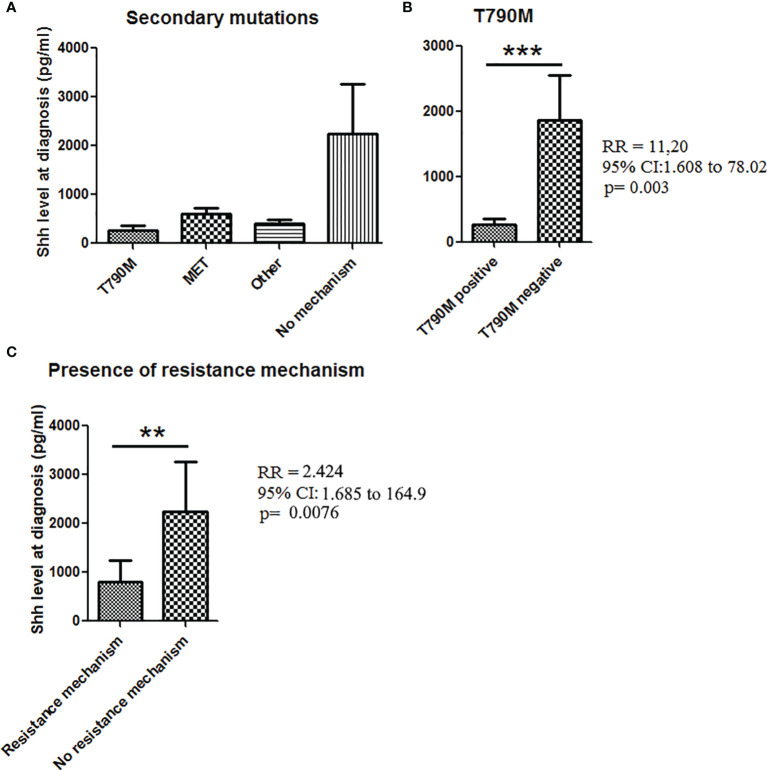
Shh concentrations in plasma from patients at diagnosis according to the presence of secondary mutations. **(A)** Patient samples analyzed for Shh expression at baseline were classified according to the progression mechanism observed at the time of the progression. **(B)** Association between Shh concentrations and the presence of T790M mutation at the time to the progression. **(C)** Shh concentration in plasma from patients according to the presence of a resistance mechanism. A Mann–Whitney test was used to analyze the differences between means. **p < 0.01; ***p < 0.001.

Finally, we tested how effective Shh levels at baseline could be related with acquired mechanisms of TKI resistance. Among the 61 patients with available Shh concentration at baseline, 35 went in progression. Analysis of the resistance mechanism was available only for 27/35 patients including 17 patients with a known resistance mechanism. The main resistance mechanisms were T790M (*n* = 9), MET amplification (*n* = 4), and other (*n* = 4) (**Figures 5A**, **S1**). Previous studies demonstrated that activation of Hedgehog pathway is a T790M-independent mechanism of EGFR-TKI resistance in *EGFR*-mutated NSCLC (23). Consistently, we observed higher Shh levels at baseline in patients (*n* = 19) with T790M-independent resistance mechanisms at the time of disease progression, whereas lower levels of Shh were observed in patients positive for T790M mutation at the time of progressive disease (with erlotinib, gefitinib, and afatinib treatments). Correlation tests confirmed the association between lower baseline levels of Shh and the acquisition of T790M mutation at the time of progression [RR (95% CI), 11.2 (1.61–78), *p* = 0.0013], supporting that higher Shh levels at baseline may be associated with EGFR-independent resistance mechanisms at the time of progression (**Figure 5B**). Interestingly, when we classified samples according to the presence of a resistance mechanism (*n* = 16) or not (11), we found higher levels of Shh in patients presenting no resistance mechanism (**Figure 5C**). This was confirmed by a positive correlation between high Shh levels at diagnosis and the absence of a resistance mechanism [RR (95% CI), 2.424 (1.68–164.9), *p* = 0.0076], suggesting that activation of hedgehog is independent not only of T790M, but also of other resistance mutations including.

## Discussion

This study provides evidence that Shh pathway activation, as assessed by high levels of plasma Shh at diagnosis, is associated with resistance to EGFR TKIs in *EGFR-*mutated NSCLC.

Because blood collection is less invasive, growing efforts have been made these past years to study the prognostic value of blood components, including circulating DNA, exosomes, and soluble proteins (30–32). Noman et al. observed that increased levels of circulating Shh are associated with a worse survival, proposing that blood Shh can stand as cancer biomarker (17). Determining whether the plasma Shh could reflect the molecular dynamics of the Hedgehog pathway within the tumor bulk remains a key challenge. Raz et al. established a strong correlation between Shh expression and downstream Hedgehog pathway effectors including Smo, Gli1, and Gli2 (27). Similarly, we clearly established that plasma levels of Shh are positively correlated with nuclear expression of Gli1 in lung tumor cells, providing evidence that plasma Shh, reflecting nuclear localization of Gli1, can be used to study the activation of Hh pathway in *EGFR*-mutated NSCLC.

Among the clinicopathological parameters influencing the survival of TKI-treated patients, we found an enrichment of Shh in plasma from young patients, male patients, and patients with multiple metastatic sites and with L858R mutations. In *EGFR*-mutated NSCLC, Shh pathway promotes stemness, epithelial-to-mesenchymal transition (EMT), invasion, and resistance to therapies (28). Bermudez et al. reported that while the endogenous Gli1/2 signaling supports autonomous proliferation of NSCLC, the secreted Shh educates the tumor microenvironment including fibroblasts, to produce Shh itself, and proangiogenic and metastatic factors (33). Accordingly, in most cancers, increased levels of Shh are linked to higher invasive capabilities and drug resistance (17, 34). Consistently, we found elevated levels of plasma Shh in patients presenting several metastatic sites (*n* > 3).

We have previously observed that high activation of Shh pathway as assessed through the IHC of Gli proteins correlated with shorter survival in patients treated with immune checkpoint inhibitors or platinum-based chemotherapy (20, 21). Gialmanis et al. also observed correlations of Hedgehog pathway activation (as assessed by IHC of Gli1 and Gli2) with histological type and unfavorable prognosis parameters in NSCLC (35). Overall, studies support a positive correlation between Hh signaling as assessed by expression of Gli1/2 and shorter OSs (21, 35–37). Bora-Singhal et al. have addressed the relation between Hedgehog signaling and the outcome of patients treated with EGFR-TKI. In their study, the authors used Gli1 expression as the surrogate of Hh activation. Consistently, they demonstrated that Hedgehog activation as assessed by the mean of Gli1 expression correlated with poor OS (36).

Our study showed that Shh was highly enriched in patients unresponsive to the treatment. In the study of Dong et al., the transcript of Gli1 was found to be positively correlated with Gefitinib IC50, when the drug was used to treat different NSCLC cell lines (10). Hh signaling is generally quite inactive in NSCLC cells responsive to EGFR-TKI, and fully operative in EGFR-TKI-resistant NSCLC (22). The role for the synergistic crosstalk between Hh and EGFR signaling in mediating EMT and drug resistance to EGFR inhibition has been established elsewhere (36–38). Hedgehog pathway, through EMT induction, leads to reduced sensitivity to EGFR-TKIs in NSCLC (39). The RAF/MEK/ERK signaling cascade upregulates Gli1 expression/activation to promote cancer cell, proliferation, survival, invasion, and drug resistance. However, even though Shh could be activated downstream EGFR signaling, the two pathways have overlapping roles, favoring EMT, metastasis, and drug resistance (40). Therefore, upon TKI-mediating EGFR inhibition, cancer cells rapidly upregulate Shh signaling to compensate for the absence of EGFR signaling (38). Concordantly, we have observed an increase of Shh levels in patients after induction therapy. *In vitro* and *in vivo* studies revealed that upon a continuous treatment with EGFR-TKI, sensitive EGFR-mutated NSCLC cells become refractory to EGFR-TKI after a period where the Hh pathway is upregulated (38, 41). Consequently, it has been observed in preclinical studies that the pharmacological interference of Hh signaling keeps cells sensitive to TKI-EGFR treatment including gefitinib, afatinib, or osimertinib. Notably, the selective Smo inhibitors such as Sonidegib, GDC-0449, and LDE225 are capable both to prevent resistance to EGFR-TKI and to restore TKI sensitivity in refractory EGFR-mutated NSCLC cells (38, 39, 42). These provide the basis to test the association of EGFR-TKI with inhibitors of Hedgehog signaling, both to prevent drug resistance and to sensitize refractory EGFR-mutated NSCLC patients (43, 44).

Putting our observations in parallel with preclinical studies, we can suggest a model where insensitive patients have higher levels of Shh that make them refractory to therapy, while responsive patients present lower levels of Shh, which will start to increase upon drug treatment, until a peak of Shh that will correspond to EGFR-independent resistance mechanism and disease progression. Therefore, the increase in Shh levels along the treatment could correspond to the emergence of resistance. Further studies should be done to (i) find the Shh cutoff levels that could accurately define responder and non-responder patients, and (ii) determine the fold increase corresponding to disease progression.

Overall, we provided the evidence that increased levels of Shh could be related to the emergence of TKI resistance, providing the rationale to implement larger studies in EGFR-mutated NSCLC treated with TKIs, with the following aims: to validate plasma levels of Shh as a new criterion of patient stratification, and to use and to validate the efficacy of Hh inhibitors in clinical studies.

## Data Availability Statement

The raw data supporting the conclusions of this article will be made available by the authors, without undue reservation.

## Ethics Statement

The studies involving human participants were reviewed and approved by CPP IDF n°8 and CPP Ouest 6. The patients/participants provided their written informed consent to participate in this study.

## Author Contributions

PTK: data collection and analyses, statistical analyses, writing of the manuscript, and revision of the manuscript. AS: data collection and analyses, statistical analyses, and revision of the manuscript. AC, CJ, J-FE, MP, and SO-C: data collection and analyses. PS: data collection, data analyses, and revision of the manuscript. EG-L: conception of the project, data analyses, data collection and analyses, statistical analyses, writing revision, and approval of the final version of the manuscript. All authors contributed to the article and approved the submitted version.

## Funding

This study received funding from Legs Poix subvention (2018). PS and SO-C are supported by funding from the Ligue Nationale contre le Cancer (Drôme and Puy-de-Dôme Committees; SOC), the National Cancer Institute (INCa PRTK-17-154; PS), Aviesan ITMO Cancer (18CN044-00; PS), the Integrated Cancer Research Site LYriCAN (INCa-DGOS-Inserm_12563). None of the funders aforementioned had any role in study design, data collection and analysis, decision to publish, or preparation of the manuscript.

## Conflict of Interest

EG-L: AstraZeneca (honoraria, advisory board, and research grant), Bristol-Myers-Squibb (honoraria, advisory board, and research grant), MSD (honoraria and advisory board); J-FE: Bristol-Myers-Squibb (advisory board); SO-C and PS: AstraZeneca (research grant).

The remaining authors declare that the research was conducted in the absence of any commercial or financial relationships that could be construed as a potential conflict of interest.

## Publisher’s Note

All claims expressed in this article are solely those of the authors and do not necessarily represent those of their affiliated organizations, or those of the publisher, the editors and the reviewers. Any product that may be evaluated in this article, or claim that may be made by its manufacturer, is not guaranteed or endorsed by the publisher.
