# Corrigendum: High Circulating Sonic Hedgehog Protein Is Associated With Poor Outcome in EGFR-Mutated Advanced NSCLC Treated With Tyrosine Kinase Inhibitors

**DOI:** 10.3389/fonc.2022.852540

**Published:** 2022-02-09

**Authors:** Paul Takam Kamga, Aurélie Swalduz, Adrien Costantini, Catherine Julié, Jean-François Emile, Maurice Pérol, Virginie Avrillon, Sandra Ortiz-Cuaran, Pierre De Saintigny, Etienne Giroux-Leprieur

**Affiliations:** ^1^ Université Paris-Saclay, UVSQ, EA 4340 BECCOH, Boulogne-Billancourt, France; ^2^ Department of Medical Oncology, Centre Léon Bérard, Lyon, France; ^3^ Univ Lyon, Université Claude Bernard Lyon 1, INSERM 1052, CNRS 5286, Centre Léon Bérard, Centre de Recherche en Cancérologie de Lyon, Lyon, France; ^4^ Department of Respiratory Diseases and Thoracic Oncology, APHP – Hopital Ambroise Pare, Boulogne-Billancourt, France; ^5^ Department of Pathology, APHP – Hopital Ambroise Pare, Boulogne-Billancourt, France

**Keywords:** non-small cell lung cancer (NSCLC), epidermal growth factor receptor (EGFR), Sonic Hedgehog (Shh), biomarker, tyrosine kinase inhibitor (TKI)

An author name was incorrectly spelled as Etienne Giroux LEPRIEUR. The correct spelling is Etienne GIROUX-LEPRIEUR.

The authors apologize for this error and state that this does not change the scientific conclusions of the article in any way. The original article has been updated.

## Publisher’s Note

All claims expressed in this article are solely those of the authors and do not necessarily represent those of their affiliated organizations, or those of the publisher, the editors and the reviewers. Any product that may be evaluated in this article, or claim that may be made by its manufacturer, is not guaranteed or endorsed by the publisher.

