# Supplementary figures and images for: High Circulating Sonic Hedgehog Protein Is Associated With Poor Outcome in EGFR-Mutated Advanced NSCLC Treated With Tyrosine Kinase Inhibitors
Source: Front Oncol. 2021 Dec 14;11:747692. doi: 10.3389/fonc.2021.747692 (PMC8712335; doi:10.3389/fonc.2021.747692)

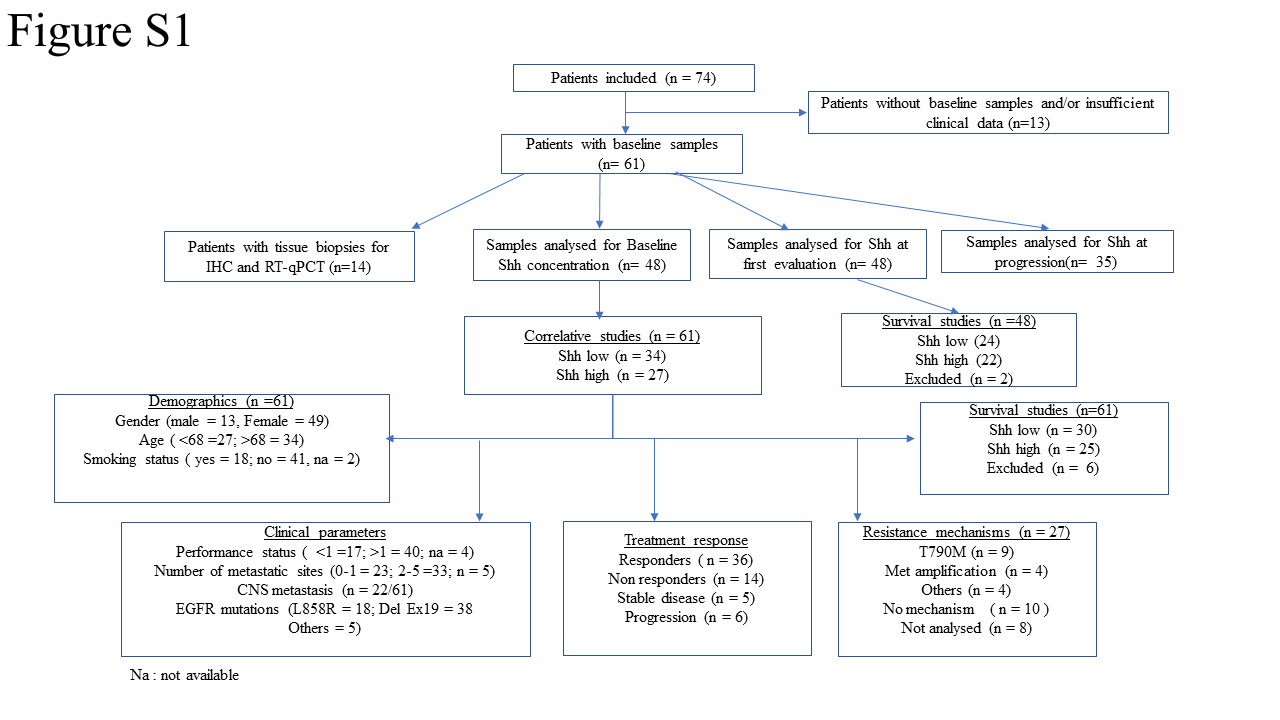

Supplement: Supplementary file 1 [file Image_1.tif]

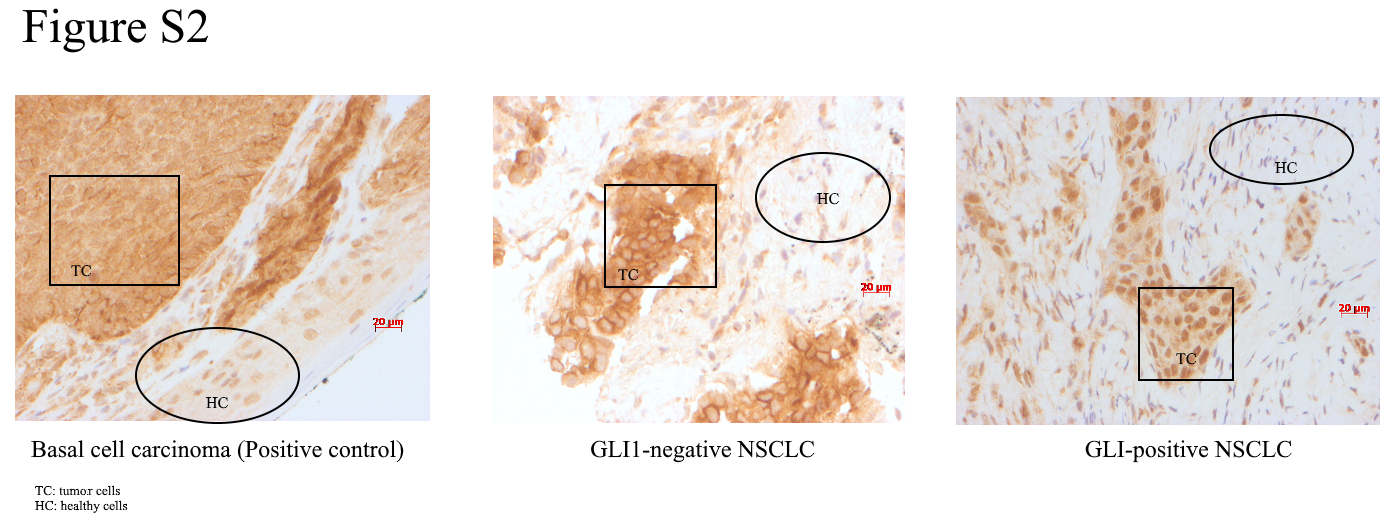

Supplement: Supplementary file 2 [file Image_2.tiff]
