# Supplementary material for: High Circulating Sonic Hedgehog Protein Is Associated With Poor Outcome in EGFR-Mutated Advanced NSCLC Treated With Tyrosine Kinase Inhibitors
Source: Front Oncol. 2021 Dec 14;11:747692. doi: 10.3389/fonc.2021.747692 (PMC8712335; doi:10.3389/fonc.2021.747692)
Supplement: Supplementary file 3 [file Table_1.docx]

**Table S1:** List of primers used to analyze the relative expression of Gli1 target genes. All primers were commercially acquired from ThermoFisher Scientifi (France).

| Genes | Reference |
| --- | --- |
| *PTCHD1* | Hs00288486_m1 |
|  |  |
| *JAG2* | Hs00171432_m1 |
|  |  |
| *HHIP* | Hs01011015_m1 |
|  |  |
| ACTB | Hs99999903_m1 |
