# Supplementary material for: High Circulating Sonic Hedgehog Protein Is Associated With Poor Outcome in EGFR-Mutated Advanced NSCLC Treated With Tyrosine Kinase Inhibitors
Source: Front Oncol. 2021 Dec 14;11:747692. doi: 10.3389/fonc.2021.747692 (PMC8712335; doi:10.3389/fonc.2021.747692)
Supplement: Supplementary file 4 [file Table_2.docx]

**Table S2:** Shh levels in the plasma of patients at diagnostic

| Statistic | Concentration (ng/ml) |
| --- | --- |
| Nb. observations | 61 |
| Minimum | 0.312 |
| Maximum | 11522.562 |
| Q1 | 203.813 |
| Q2 (Median) | 466.185 |
| Q3 | 765.803 |
| IQR | 561.99 |
| T1 | 233.4 |
| T2 | 551.7 |
| Mean | 1041.223 |
| SD (n-1) | 1972 |
| Normality Test (KS test) | p < 0.001 |
