# Supplementary material for: High Circulating Sonic Hedgehog Protein Is Associated With Poor Outcome in EGFR-Mutated Advanced NSCLC Treated With Tyrosine Kinase Inhibitors
Source: Front Oncol. 2021 Dec 14;11:747692. doi: 10.3389/fonc.2021.747692 (PMC8712335; doi:10.3389/fonc.2021.747692)
Supplement: Supplementary file 5 [file Table_3.docx]

**Table S3. SHH expression levels and demographics:** Patients samples analyzed for Shh expression were classified according to clinicopathological features.

| Parameters | | Shh levels (pg/mL) (mean ± SEM) | P-value  (t-test) | Relative Risk (95% CI) | P-value |
| --- | --- | --- | --- | --- | --- |
| Patients | N = 61 | 1041.223 ± 252.5 |  | | |
| Gender | Females | 692.0 ± 156. | 0.0044 | 1.102 (0.8456 - 1.436) | 0.2594 |
|  | Males | 1678 ± 744.5 |  |  |  |
| Age | <68 | 1293 ± 397.0 | 0.0220 | 0.9643 (0.4848 - 1.918) | 0.9643 |
|  | 67 > | 518.0 ± 89.69 |  |  |  |
| Smokers (57/61) | Never (34) | 1067 ± 274.0 | 0.0479 | 0.9908(0.6776 - 1.449) | 0.6057 |
|  | Previous /Current | 335.3 ± 50.03 |  |  |  |
| Number of Metastatic sites | [0-2] | 576.2 ± 156.7 | 0.0416 | 1.117 (0.7185 - 1.737) | 0.3103 |
|  | [3-5] | 1292 ± 428.7 |  |  |  |
| CNS metastasis | Yes | 872.6 ± 284.1 | 0.4500 | 0.9244 (0.5780 - 1.478) | 0.4773 |
|  | No | 927.4 ± 321.5 |  |  |  |
| Performance status | [0] | 514.6 ± 59.89 | 0,1234 | 1.143 (0.4852- 2.692) | 0.7595 |
|  | [1-2] | 1121 ± 299.8 |  |  |  |
