# Supplementary material for: High Circulating Sonic Hedgehog Protein Is Associated With Poor Outcome in EGFR-Mutated Advanced NSCLC Treated With Tyrosine Kinase Inhibitors
Source: Front Oncol. 2021 Dec 14;11:747692. doi: 10.3389/fonc.2021.747692 (PMC8712335; doi:10.3389/fonc.2021.747692)
Supplement: Supplementary file 6 [file Table_4.docx]

| Parameters | | OS (days) | HR (95% CI); p-value | PFS (days) | HR (95% CI), p-value |
| --- | --- | --- | --- | --- | --- |
| Gender | Females (53) | 1098 | 1.215 (0.3248 -4.541); p = 0.77 | 429 | 1.99 (0.7365- 5.428); p = 0.17 |
|  | Males (14) | Undefined |  | 255 |  |
| Age | <68 (26) | Undefined | 0.287 (0.11- 0.723); p = 0.0090 | 541 | 0.515 (0.277 – 0.956); p = 0.0354 |
|  | 67 > (28) | 884 |  | 254 |  |
| Smokers | Never (45) | Undefined | 0.786 (0.309 - 1.998); p = 0.61 | 326 | 1.11 (0.603 – 2.043); p = 0.73 |
|  | Previous /Current (22) | 1074 |  | 428 |  |
| Del19 | Positive (36) | Undefined | 0.28 (0.1- 0.785); p = 0.0156 | 428 | 0.7993 (0.4229 -1.511); p = 0.49 |
|  | Negative (25) | 879 |  | 309 |  |
| L858R | Positive (20) | 879 | 2.496 (0.866 - 7.191); p = 0.09 | 309 | 1.398 (0.721 – 2.711); p = 0.322 |
|  | Negative (41) | Undefined |  | 428 |  |
| Number of Metastatic sites | [0-2] (43) | 1098 | 1.239 (0.388 - 3.955); p = 0.717 | 455 | 0.595 (0.286 – 1.239) |
|  | [3-5] (21) | Undefined |  | 266 |  |
| CNS metastasis (n = 67) | Yes (31) | Undefined | 0.925 (0.354 – 2.419); p = 0.87 | 318 | 1.193 (0.661 – 2.151); p = 0.559 |
|  | No (22) | 1098 |  | 326 |  |
| Performance status (n = 67) | [0] (15) | Undefined | 06.168 (0.1838 - 2.070); p = 0.434 | 317.5 | 0.9891 (0.495 – 1.978) |
|  | [1-2] (47) | 1074 |  | 428 |  |

**Table S4. Survival pattern of the 74 patients according to the clinicopathological parameter.** Kaplan-Meir and log-rank test were used to study survival in each specific group.
